# Toll-like receptor pre-stimulation protects mice against lethal infection with highly pathogenic influenza viruses

**DOI:** 10.1186/1743-422X-8-97

**Published:** 2011-03-04

**Authors:** Kyoko Shinya, Tadashi Okamura, Setsuko Sueta, Noriyuki Kasai, Motoko Tanaka, Teridah E Ginting, Akiko Makino, Amie J Eisfeld, Yoshihiro Kawaoka

**Affiliations:** 1 Department of Microbiology and Infectious Diseases, Graduate School of Medicine, Kobe University, Kobe 650-0017, Japan; 2 Institute for Animal Experimentation, Tohoku University Graduate School of Medicine, Sendai 980-8575, Japan; 3 Department of Pathobiological Sciences, School of Veterinary Medicine, University of Wisconsin-Madison, Madison, Wisconsin 53706, USA; 4 Division of Virology, Institute of Medical Science, University of Tokyo, Tokyo 108-8639, Japan ERATO Infection-Induced Host Responses Project, Japan Science and Technology Agency, Saitama 332-0012, Japan

## Abstract

Since the beginning of the 20th century, humans have experienced four influenza pandemics, including the devastating 1918 'Spanish influenza'. Moreover, H5N1 highly pathogenic avian influenza (HPAI) viruses are currently spreading worldwide, although they are not yet efficiently transmitted among humans. While the threat of a global pandemic involving a highly pathogenic influenza virus strain looms large, our mechanisms to address such a catastrophe remain limited. Here, we show that pre-stimulation of Toll-like receptors (TLRs) 2 and 4 increased resistance against influenza viruses known to induce high pathogenicity in animal models. Our data emphasize the complexity of the host response against different influenza viruses, and suggest that TLR agonists might be utilized to protect against lethality associated with highly pathogenic influenza virus infection in humans.

## Background

During the 20th century, humans experienced three influenza pandemics, each resulting in significant global mortality: 20 to 40 million deaths in 1918 (Spanish influenza), 1 to 4 million deaths in 1957 (Asian influenza), and 1 to 4 million deaths in 1968 (Hong Kong influenza) [1]. In addition, the pandemic (H1N1) 2009 virus has spread rapidly around the world since spring of 2009; and HPAI viruses have been circulating worldwide since late 2003. Although HPAI viruses are not yet efficiently transmitted to or among humans, their sustained proliferation and continued genetic evolution in avian species, combined with parallel infections in humans, makes this an eventual possibility.

Some patients infected with either 2009 pandemic H1N1 or HPAI viruses develop acute respiratory distress syndrome and severe alveolar damage [2-5]. This pathologic condition is associated with a strong upregulation of cytokines and chemokines: in particular, interferon-induced protein 10 (IP-10; CXCL10), monokine induced by interferon gamma (MIG; CXCL9), monocyte chemotactic protein 1 (MCP-1; CCL2), interleukin (IL)-8, IL-10, IL-6, interferon γ (IFN-γ), and tumor necrosis factor α (TNF-α) [6-10]. In the macaque model, infection with the 1918 Spanish influenza virus markedly increased serum levels of IL-6, IL-8, MCP-1, and RANTES (RANTES; CCL5) [11]. Thus, it has been suggested that the severity of influenza is associated with the aberrant induction of innate immunity.

Pre-stimulation of innate immunity has been shown to confer resistance against lethal influenza infection. Specifically, influenza A virus titers decreased in cells pre-treated with TNF-α, and inoculation of mice with bacterial lysates before viral infection protects against lethal influenza pneumonia [12-15]. Moreover, the general stimulation of innate immunity with interferon α, as well as the stimulation of specific Toll-like receptors (TLRs), promotes survival in mouse models of lethal influenza pneumonia [16-19]. However, the ability of innate immunity pre-stimulation to attenuate disease associated with HPAI viruses has not been explored. In the present study, we aimed to determine the protective effects of TLR pre-stimulation in mice inoculated with influenza A viruses.

## Materials and methods

### Cell lines

Madin-Darby canine kidney (MDCK) cells were maintained in Dulbecco's modified Eagle's medium (DMEM) supplemented with 10% fetal calf serum, and 293T human embryonic kidney cells were maintained in minimal essential medium (MEM) with 5% newborn calf serum. All cells were maintained at 37°C in an atmosphere of 5% CO_2_.

### Viruses

Influenza A/Puerto Rico/8/34 (PR8; H1N1) and A/Vietnam/1203/04 (VN1203; H5N1) stock viruses were prepared in 10-day-old embryonated chicken eggs or MDCK cells, respectively. After MDCK cells were inoculated with influenza virus, they were grown in MEM containing 0.3% BSA with TPCK-trypsin (0.5 ug/ml) to propagate PR8 or without TPCK-trypsin to propagate VN1203. One reassortant virus was generated from plasmids by reverse genetics, as described previously [20,21]. The reassortant possessed the hemagglutinin (HA) segment from influenza A/South Carolina/1918 (H1N1) and the remaining seven viral RNA segments from influenza A/WSN/33 (H1N1), and was designated SpHA/WSN. The SpHA/WSN transfectant produced in 293T cells were used to inoculate MDCK cells for stock virus production. Stock virus titers were determined by the median egg infectious dose (EID_50_) or plaque assay. Experiments using VN1203 or SpHA/WSN were conducted in an enhanced biosafety level 3 (BSL3+) containment laboratory approved for such use by the Centers for Disease Control and Prevention and the United States Department of Agriculture.

### Lethal dose studies in mice

BALB/c mice (6-week-old) used in this study were maintained in a specific pathogen-free environment. All manipulations (TLR pre-treatments and virus inoculations) were performed in mice anesthetized with sevoflurane. Pre-treatments with the indicated TLR ligands were carried out by intranasal administration of 100 μl phosphate-buffered saline (PBS) containing these ligands at the indicated times before infection. Control mice were inoculated with PBS only. To determine the mouse lethal dose 50 (MLD_50_) following pre-stimulation, anesthetized BALB/c mice were intranasally inoculated with 10-fold serial dilutions of virus in 50 μl PBS (each group, n = 3). Mice were monitored daily over 14 days for disease symptoms and survival, and the MLD_50 _value was calculated according to the method of Reed and Muench [22]. To minimize the number of animals used for these experiments, we performed each MLD_50 _titration once. Animal care and experimental procedures were approved by the Animal Research Committees at Tohoku University and the University of Wisconsin-Madison.

### TLR ligand pre-treatment in mice

For initial MLD_50 _determination with PR8, we used LPS from *Escherichia coli *(*E. coli*) serotype O26:B6 (Sigma, Tokyo; catalog #L8274) at 2.5, 1.25, 0.625, 0.3125, or 0.15625 mg/kg. For subsequent MLD_50 _experiments comparing the antiviral effects of stimulating different TLRs, we used the following TLR ligands from InvivoGen (San Diego, CA, USA): synthetic mycoplasmal lipoprotein (FSL-1, 50 μg/kg; cat# tlrl-fsl) as the TLR2 ligand; analog of dsRNA (Poly(I:C), 1.25 mg/kg; cat# tlrl-pic) as the TLR3 ligand; LPS from *E. coli *K12 msbB strain (2.5 mg/kg; cat# tlrl-mklps) and LPS from *E. coli *serotype O111:B4 (2.5 mg/kg; cat# tlrl-pelps) as TLR4 ligands; and a guanosine analog (loxoribine, 2.5 mg/kg; cat# tlrl-lox) as the TLR7 ligand.

## Results

### TLR4 prestimulation with lipopolysaccharide protects mice against lethal influenza virus challenge

Mice pretreated with a bacterial cell lysate, which induced robust inflammation likely through LPS-dependent activation of the TLR4 pathway, were more likely to survive a lethal influenza virus challenge [15]. Similarly, specific activation of the TLR4 pathway with synthetic agonists protected mice against influenza-induced lethality [17]. To establish our experimental system of pre-stimulation with the well-characterized TLR4 ligand LPS, we intranasally pre-treated BALB/c mice with LPS (*Escherichia coli *serotype O26:B6; 2.5, 1.25, 0.625, 0.3125, or 0.15625 mg/kg) at various times (12 or 24 hours, or 3 or 7 days) before exposure to influenza virus. LPS-treated mice were then infected with PR8, and the MLD_50 _was calculated for each experimental group. Results were expressed as the difference between the MLD_50 _of each treatment group and that of a control group (Table 1). Although some protection was afforded by LPS pre-treatment at all time points, the most effective pre-treatment time was 3 days before infection, when all LPS dosages increased the MLD_50 _by at least 10-fold. Overall, pre-treatment with 1.25 mg/kg LPS at 3 days before infection provided the best protection.

**Table 1:** Antiviral effect of intranasal prestimulation by lipopolysaccharide against influenza A/Puerto Rico/8/34.

**LPS (mg/kg) **^**a**^	Pretreatment time	**Ratio of MLD**_**50 **_**(log**_**10**_**EID**_**50**_**) between LPS-pretreated and mock-treated mice**^**b**^
0.15625	7 days	0
	3 days	1.0
	24 hrs	0
	12 hrs	0
0.3125	7 days	0.6
	3 days	1.26
	24 hrs	0
	12 hrs	0
0.625	7 days	1.0
	3 days	1.6
	24 hrs	1.0
	12 hrs	0.3
1.25	7 days	0
	3 days	1.94
	24 hrs	0.94
	12 hrs	0.94
	6 hrs	0.24
2.5	7 days	1.0
	3 days	1.3
	24 hrs	1.0
	12 hrs	0

^a^*Escherichia coli *(serotype O26:B6)-derived lipopolysaccharide (LPS), MLD_50 _= 36.4 (mg/kg)

^b ^"0" would be no difference and "1.0" would indicate that 10 times more virus is need to kill 50% of infected animals when treated with the indicated amount of LPS.

### Protective effects of pre-stimulation of various TLRs

Several reports have indicated that mice intranasally pre-treated with TLR ligands other than those for TLR4 exhibit enhanced survival when challenged with influenza virus [18,19,23]. We therefore compared several different TLR ligands to determine which would best protect against a lethal influenza virus challenge. We evaluated the effects of pre-stimulation of surface-expressed TLRs (TLR2 and TLR4) and endosomal TLRs (TLR3 and TLR7) using the following ligands: synthetic mycoplasmal lipoprotein (FSL-1, 50 μg/kg) for TLR2; synthetic analog of dsRNA (Poly(I:C); 1.25 mg/kg) for TLR3; LPS from *E. coli *K12 msbB strain (2.5 mg/kg) for weak TLR4 stimulation; purified LPS from *E. coli *serotype O111:B4 (2.5 mg/kg) for normal TLR4 stimulation; and the guanosine analog loxoribine (2.5 mg/kg) for TLR7. Mice pre-treated with TLR-specific ligands were challenged 3 days later with serial dilutions of PR8, and the difference in MLD_50 _was calculated for each experimental group and compared to PBS-treated controls (Table 2). As expected, all TLR ligands protected against lethal influenza pneumonia, but pre-stimulation with FSL-1 (TLR2 ligand) or msbB LPS (weak TLR4 agonist) provided the best protection, both producing a 10-fold increase in MLD_50_. These findings demonstrate that TLR4 pre-stimulation is more effective using a weak TLR4 agonist, and support the hypothesis that lung cells express both TLR2 and TLR4 [24].

**Table 2:** Antiviral effect of prestimulation with Toll-like receptor specific ligands against influenza A/Puerto Rico/8/34.

TLR ligand	Receptor	**Ratio of MLD**_**50 **_**(log**_**10**_**EID**_**50**_**) between LPS-pretreated and mock-treated mice**
Synthetic mycoplasmal lipoprotein (FSL-1)	TLR2	1.0
Synthetic analog of dsRNA (Poly(I:C))	TLR3	0.66
LPS from *E. coli *K12 msbB	TLR4	1.0
LPS from *E. coli *serotype O111:B4		0.66
Guanosine analog (loxoribine)	TLR7	0.66

### Differential effects of TLR prestimulation on lethal pneumonia associated with highly pathogenic influenza viruses in mice

We hypothesized that TLR prestimulation-mediated protection against lethal infection by a mouse-adapted influenza strain could extend to influenza virus strains that induce high pathogenicity. To test this hypothesis, we infected mice with or without either TLR2 or TLR4 pre-stimulation with an HPAI H5N1 virus isolate (VN1203) or a reassortant virus possessing the hemagglutinin (HA) gene segment from the 1918 Spanish influenza virus (SpHA/WSN) (Table 3). The high pathogenicity of these virus strains was previously demonstrated in mice [20,25].

**Table 3:** Antiviral effect of prestimulation by Toll-like receptor-specific ligands.

TLR stimulation	Virus strain	**Ratio of MLD**_**50 **_**(log**_**10**_**EID**_**50**_**) between LPS-pretreated and mock-treated mice**
TLR2 ^a^	A/Vietnam/1203/04	0
TLR4 ^b^	A/Vietnam/1203/04	1.0
TLR2	Sp HA/WSN ^c^	0.76
TLR4	Sp HA/WSN ^c^	0.49

^a^Synthetic mycoplasmal lipoprotein (FSL-1)

^b^LPS of *E. coli *K12 msbB (LPS agonist)

^c^Reassortant possessing the HA segment from influenza A/South Carolina/1918 (H1N1) and the remaining seven viral RNA segments from A/WSN/33 (H1N1).

We observed a difference in the ability of TLR2 and TLR4 pre-stimulation to protect against highly pathogenic viruses. Consistent with protection against PR8 in LPS-pretreated mice, TLR4 stimulation with msbB LPS (weak agonist) protected against lethality induced by VN1203 (10-fold increase in MLD_50 _compared to untreated control). In contrast, TLR2 stimulation with FSL-1 did not protect against VN1203 (Table 3). However, we observed the opposite pattern with SpHA/WSN: TLR2 pre-stimulation conferred better protection against lethality compared to TLR4 pre-stimulation. These results suggest that the best method of stimulating innate immunity depends on the particular virus in question.

## Conclusions

Our results indicate that TLR pre-stimulation protects mice from lethal challenge with highly pathogenic influenza viruses. Although previous reports have suggested the efficacy of TLR pre-stimulation in promoting survival after influenza challenge[15,17], we provide the first evidence that TLR activation is effective against HPAI and other highly pathogenic influenza strains.

Inactivated H5N1 virus has been shown to induce acute lung injury through the TLR4-TRIF pathway [26], suggesting that viral surface glycoproteins HA and/or neuraminidase (NA), may be sensed by TLR4 on the cell surface. TLR4 responds to molecular signatures of microbial origin (i.e., LPS), but more recent evidence suggests that TLR4 can also be activated by viral infection [27-29]. Thus, robust replication and expression of viral antigens may induce hyperactivation of the TLR4 signaling pathway and lead to lung injury; the H5N1 virus is not likely to be an exception. Our data showed that TLR4 pre-stimulation could also offer protection against lethal HPAI infections. It is interesting that a molecule associated with influenza severity can also protect against influenza-induced lethality when given prophylactically.

While pre-stimulation of both TLR2 and TLR4 signaling pathways protected against the PR8 virus, protection against the VN1203 virus could only be achieved by TLR4 pre-stimulation. Contrastingly, a virus carrying the 1918 Spanish influenza HA molecule was not inhibited by a TLR4 agonist, but rather was more affected by pre-stimulation of TLR2. TLR2 and TLR4 induce overlapping and unique signaling associated with innate immunity, and these pathways may be differentially required for protection against different influenza viruses. We suggest that TLR4-mediated signaling may have a principle role in protection against HPAI viruses, while control of Spanish influenza may involve TLR2-related mechanisms. Our data highlights the complex nature of innate signaling in response to infection with different influenza virus strains, and emphasizes the need for further dissection of the pathways that are involved in controlling influenza infection and promoting influenza pathogenesis. Further, we suggest that this information could be utilized in the development of countermeasures against highly pathogenic influenza virus infections in humans.

## Competing interests

The authors declare that they have no competing interests.

## Authors' contributions

KS and YK conceived and designed the experiments. KS, TO, SS, NK, MT, TEG, and AK performed the experiments. KS, AK, AJE, and YK analyzed the data and wrote the paper. All authors read and approved the final manuscript.
